# Antigenicity and immune correlate assessment of seven *Plasmodium falciparum* antigens in a longitudinal infant cohort from northern Ghana

**DOI:** 10.1038/s41598-019-45092-4

**Published:** 2019-06-13

**Authors:** Kwadwo Asamoah Kusi, Joao Aguiar, Selassie Kumordjie, Felix Aggor, Jessica Bolton, Andrea Renner, Eric Kyei-Baafour, Naiki Puplampu, Maria Belmonte, Daniel Dodoo, Ben Adu Gyan, Michael Fokuo Ofori, Abraham Rex Oduro, Frank Atuguba, Kwadwo Ansah Koram, Nehkonti Adams, Andrew Letizia, Eileen Villasante, Martha Sedegah

**Affiliations:** 1 Immunology Department, Noguchi Memorial Institute for Medical Research, College of Health Sciences, University of Ghana, Legon, Ghana; 2 Malaria Department, Naval Medical Research Center, Silver Spring, MD 20910 USA; 3 US Naval Medical Research Unit No. 3, Ghana Detachment, Accra, Ghana; 4 Navrongo Health Research Centre, Ghana Health Service, Navrongo, Ghana; 5 Epidemiology Department, Noguchi Memorial Institute for Medical Research, College of Health Sciences, University of Ghana, Legon, Ghana; 6 Present Address: University of Pittsburgh School of Medicine, Division of Rheumatology and Clinical Immunology, 200 Lothrop St., SBST 737, Pittsburgh, PA 15213 USA; 7 Present Address: Congressionally Directed Medical Research Programs (CDMRP) The United States Army Medical Research and Materiel Command (USAMRMC), 1054 Patchel Street, Fort Detrick, MD 21702 USA; 8 Present Address: Navy Medicine Professional Development Center, Academic Programs Directorate, 8955 Wood Road, Bldg 1, Bethesda, MD 20889 USA

**Keywords:** Malaria, Malaria

## Abstract

The current global malaria control and elimination agenda requires development of additional effective disease intervention tools. Discovery and characterization of relevant parasite antigens is important for the development of new diagnostics and transmission monitoring tools and for subunit vaccine development. This study assessed the natural antibody response profile of seven novel *Plasmodium falciparum* pre-erythrocytic antigens and their potential association with protection against clinical malaria. Antigen-specific antibody levels in plasma collected at six time points from a longitudinal cohort of one-to-five year old children resident in a seasonal malaria transmission area of northern Ghana were assessed by ELISA. Antibody levels were compared between parasite-positive and parasite-negative individuals and the association of antibody levels with malaria risk assessed using a regression model. Plasma antibody levels against five of the seven antigens were significantly higher in parasite-positive children compared to parasite-negative children, especially during low transmission periods. None of the antigen-specific antibodies showed an association with protection against clinical malaria. The study identified five of the seven antigens as markers of exposure to malaria, and these will have relevance for the development of disease diagnostic and monitoring tools. The vaccine potential of these antigens requires further assessment.

## Introduction

As malaria cases continue to decline globally, identification and characterization of *Plasmodium* parasite proteins becomes more essential to the development of integrated tools for control and prevention. To date, only a fraction of the over 5,500 proteins encoded by the *P. falciparum* genome during its multistage life cycle have been described and immunologically evaluated^1^. There is an urgent need to enrich the pipeline of vaccine development with novel candidates since an effective vaccine is one of the integrated tools that is needed to potentially ensure eradication^2^. Additionally, further characterization and understanding of human immune responses to malarial antigens will enhance eradication efforts through the development of more sensitive diagnostic^3^ and transmission monitoring^4–7^ tools. Naturally induced antigen-specific antibody levels are generally known to be influenced by such factors as host age, frequency of exposure to parasites as well as the extent of disease transmission^8^. Naturally acquired immunity to malaria has mostly been associated with antibody levels against certain parasite antigens, especially in adults repeatedly exposed to parasites^1,9,10^. It is also known that individuals living in areas of high malaria transmission usually have high levels of malaria antigen-specific antibodies compared to those in low transmission areas^8,11^. These assertions collectively point to the need for repeated or persistent parasite exposure in order to achieve the semi-immune status that has been well described among adults living in malaria endemic areas. In many instances, levels of the same antigen-specific antibodies in children have been identified to be mere markers of exposure to the parasite^6,12^ and not a marker for immunity. The dynamics of antibody levels, longevity and specificity based on the above parameters determines the usefulness and applicability of the corresponding antigen targets, either for vaccine development, diagnostics or as biomarkers for monitoring disease transmission.

While many asexual blood stage parasite antigens have been described and immunologically characterized, only a handful of pre-erythrocytic (PE) stage antigens have been identified and similarly characterized^13^. PE antigens however remain very important candidates for vaccine development^14,15^ and transmission monitoring tools^6,16^. Additionally, parasite antigens that occur in both the pre-erythrocytic and erythrocytic parasite stages are ideal multi-stage options for vaccine development. On-going work at the Naval Medical Research Center (NMRC) Malaria Department seeks to identify novel PE antigens using samples from subjects and animals immunized with radiation attenuated sporozoites (RAS) and assessing their immunogenicity for the purpose of sub-unit vaccine development. A number of such antigens have been identified and successfully expressed as recombinant proteins using a cell-free wheat-germ system^17–19^. The antigens are recognized by sera and T cells from RAS-immunized subjects^17^. Although the antigens were selected on the basis of being expressed in sporozoite and liver PE stages, localization studies with polyclonal sera induced in either rabbits or mice show that some of the antigens are concurrently expressed in asexual blood stages of the parasite^17^, suggesting that they might serve as targets of multi-stage anti-malarial immunity. The aim of this study was to assess the levels and kinetics of naturally induced antibodies to seven of these antigens in plasma from children living in an area of northern Ghana with marked seasonal malaria transmission. The seven selected antigens have been putatively described and include a glideosome-associated protein (Pf02), a transcription initiation factor TFIID subunit (Pf26), a GPI-anchored micronemal antigen (Pf56), a conserved *Plasmodium* protein with unknown function (Pf61), a cysteine protease inhibitor (Pf106), a subpellicular microtubule protein (Pf116) and a gamete egress and sporozoite traversal protein (Pf144). These were selected from a panel of 21 proteins previously expressed and characterized using sera from RAS-immunized subjects and from animal immunizations^17^.

## Results

### Participant clinical characteristics

A total of 288 plasma samples from 48 child subjects (six plasma samples per child) were included in this study. Children at each sampling time point were categorized either on the basis of having blood film parasites or having suffered a clinical malaria episode (Table 1). Over the six sampling time points, the majority of the 48 children had blood film parasites, ranging from 66.7% in May 2005 to 91.7% in September 2004. In contrast, only a limited proportion of these (4.2% at each of three different sampling time points and 8.3% in May 2005) resulted in clinical malaria episodes (Table 1).

**Table 1:** Clinical characteristics of study participants.

Clinical features	Sampling time points					
	July 2004	Sept. 2004	Nov. 2004	Jan. 2005	March 2005	May 2005
**Parasitaemia**						
Yes	43 (89.5)	44 (91.7)	41 (85.4)	37 (77.1)	36 (75.0)	32 (66.7)
No	5 (10.5)	4 (8.3)	7 (14.6)	11 (22.9	12 (25.0)	16 (33.3)
**Clinical malaria**						
Yes	0	2 (4.2)	2 (4.2)	2 (4.2)	3 (6.3)	4 (8.3)
No	48 (100)	46 (95.8)	46 (95.8)	46 (95.8)	45 (93.7)	44 (91.7)
**Anaemia**						
severe	0	2 (4.2)	1 (2.4)	0	0	0
moderate	24 (50.0)	17 (35.4)	18 (37.5)	6 (12.5)	18 (37.5)	5 (10.4)
mild	10 (20.8)	16 (33.3)	14 (29.2)	15 (31.3)	12 (25.0)	12 (25.0)
No	14 (29.2)	14 (29.2)	15 (31.3)	27 (56.3)	18 (37.5)	31 (64.6)

Data reported as absolute number (% of total number). Parasitaemia was estimated by microscopic examination of blood films.

A good proportion of children had either mild (Haemoglobin (Hb) concentration 10.0–10.9 g/dl) or moderate (Hb concentration 7.0–9.9 g/dl) anaemia over the study period, and only three cases of severe anaemia (Hb concentration <7.0 g/dl) were identified, occurring in September and November 2004 (Table 1). Overall, the number of anaemic children was highest during the rainy season and lowest during the dry season, while the opposite was true for the number of non-anaemic children.

**Figure 1:**
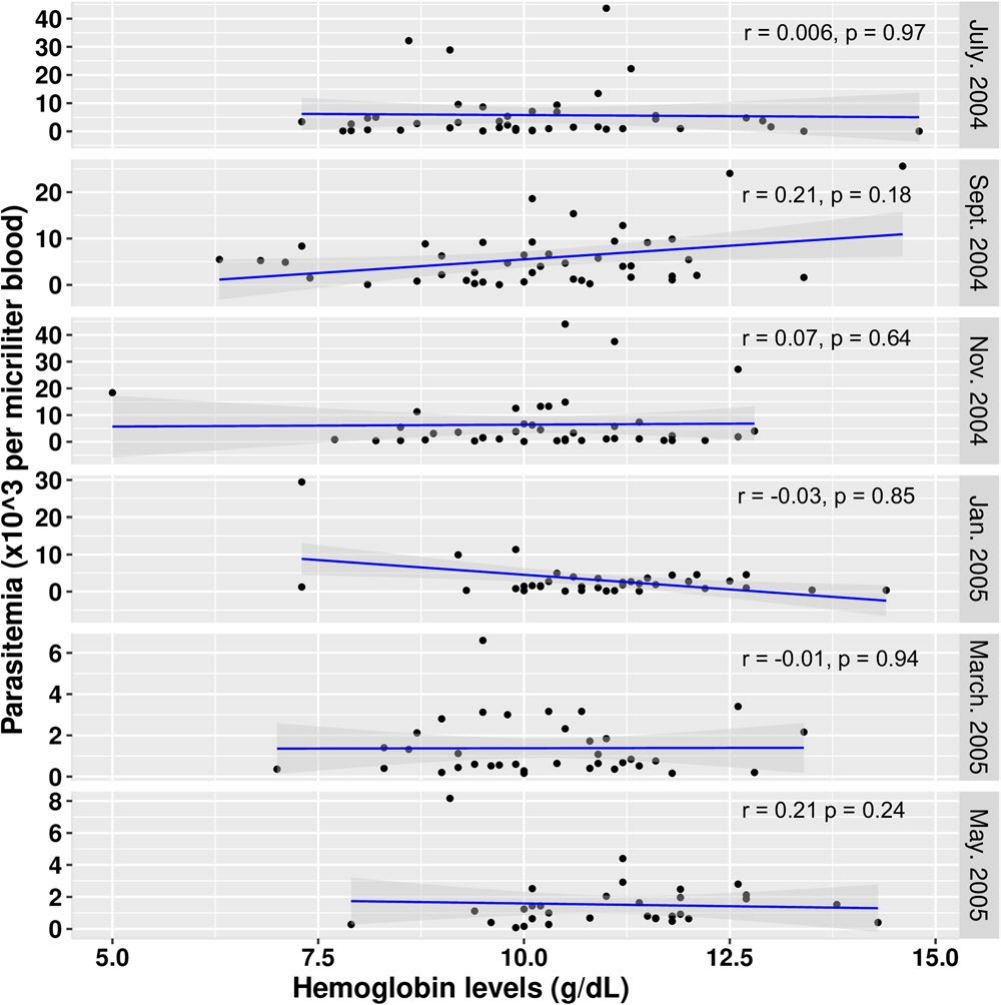
Correlation between Hb levels and parasitaemia at the different sampling time points. Correlation coefficient (r) estimates from Spearman correlation tests.

Parasitaemia was generally highest from July 2004 to January 2005 and lowest in March and May 2005, and this directly reflected the extent of malaria transmission over the period. Per sampling time point analysis showed no significant association between Hb levels and parasitaemia levels at any of the sampling time points (Fig. 1). Per sampling time point correlation analysis however showed a moderate significant negative correlation between anti-Pf116 levels and parasitaemia in November 2004 (r = −0.43, p = 0.04); all other antibody parasitaemia correlations did not show statistical significance.

### Infected individuals have increased levels of antibodies to some PE antigens

**Figure 2:**
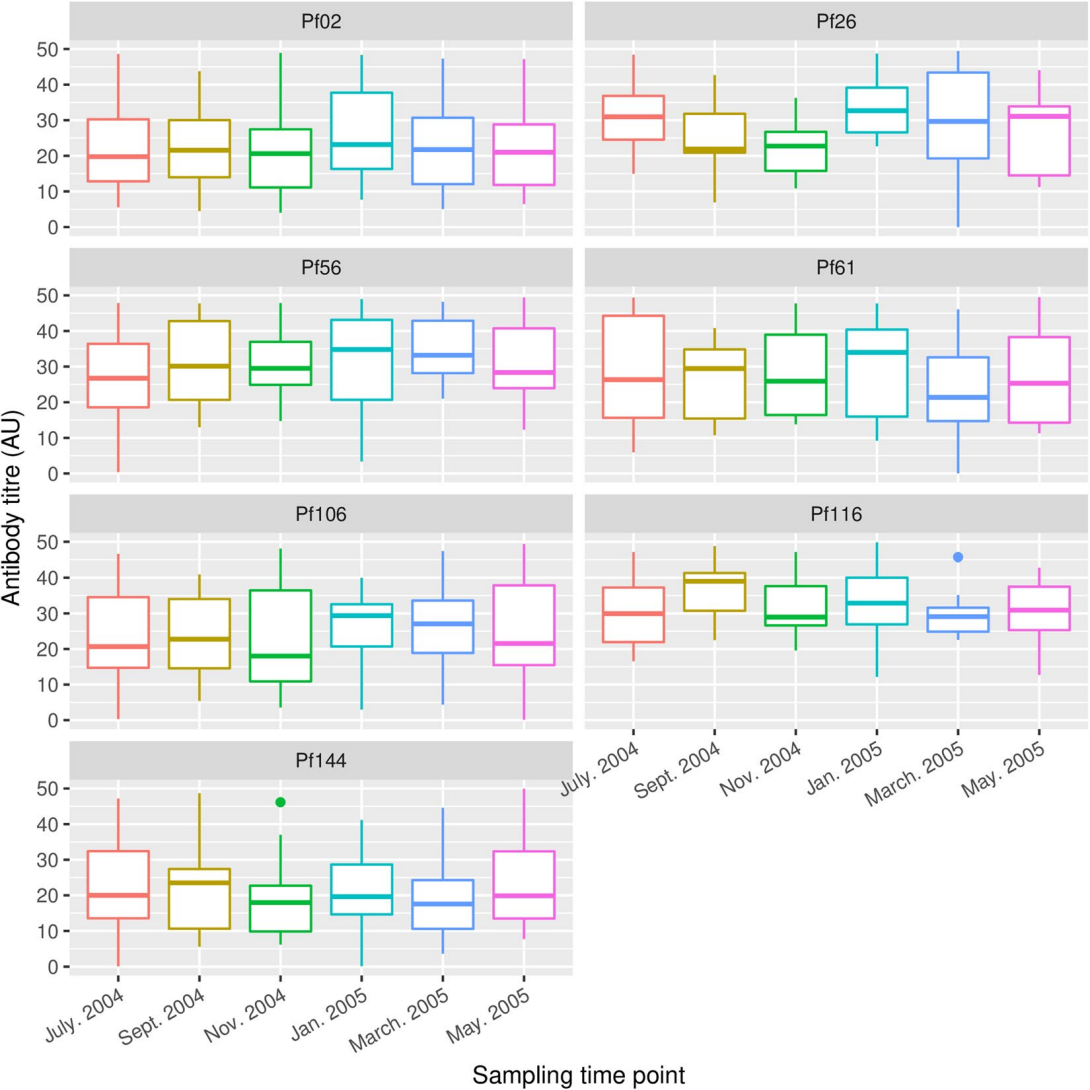
Antigen-specific antibody profiles over the six sampling time points. Boxes represent the median, 25^th^ and 75^th^ percentiles and whiskers represent 1.5 times the interquartile range. The median, 25^th^ and 75^th^ percentiles, and corresponding P values, have been provided in Supplementary Table 1.

For each of the seven PE antigens, specific antibody levels did not differ significantly when data from all study subjects were compared across the six sampling time points (Fig. 2, Supplementary Table S1).

**Figure 3:**
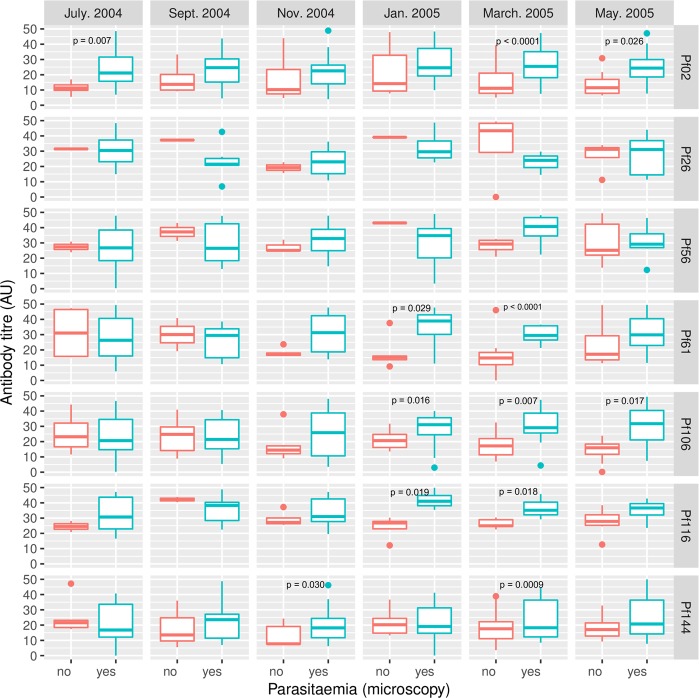
Comparison of antigen-specific antibody levels between parasitemic and non-parasitemic children at the different sampling times. Parasitaemia was determined by microscopic examination of thick and thin blood smears. Boxes represent the median, 25^th^ and 75^th^ percentiles and whiskers represent 1.5 times the interquartile range. The median, 25^th^ and 75^th^ percentiles, and corresponding P values, have been provided in Supplementary Table 2.

Antibody levels against five of the seven antigens (Pf02, Pf61, Pf106, Pf116 and Pf144) were however statistically significantly higher in subjects who had blood film parasites compared to levels in those without parasites, especially over the dry season when malaria transmission intensity was lowest (Fig. 3, Supplementary Table S2). Antibodies against the PE antigens Pf26 and Pf56 showed a similar trend of higher levels in parasitaemic children compared to non-parasitaemic children in the dry season (March 2005) although differences here did not reach statistical significance (Supplementary Table S2).

**Figure 4:**
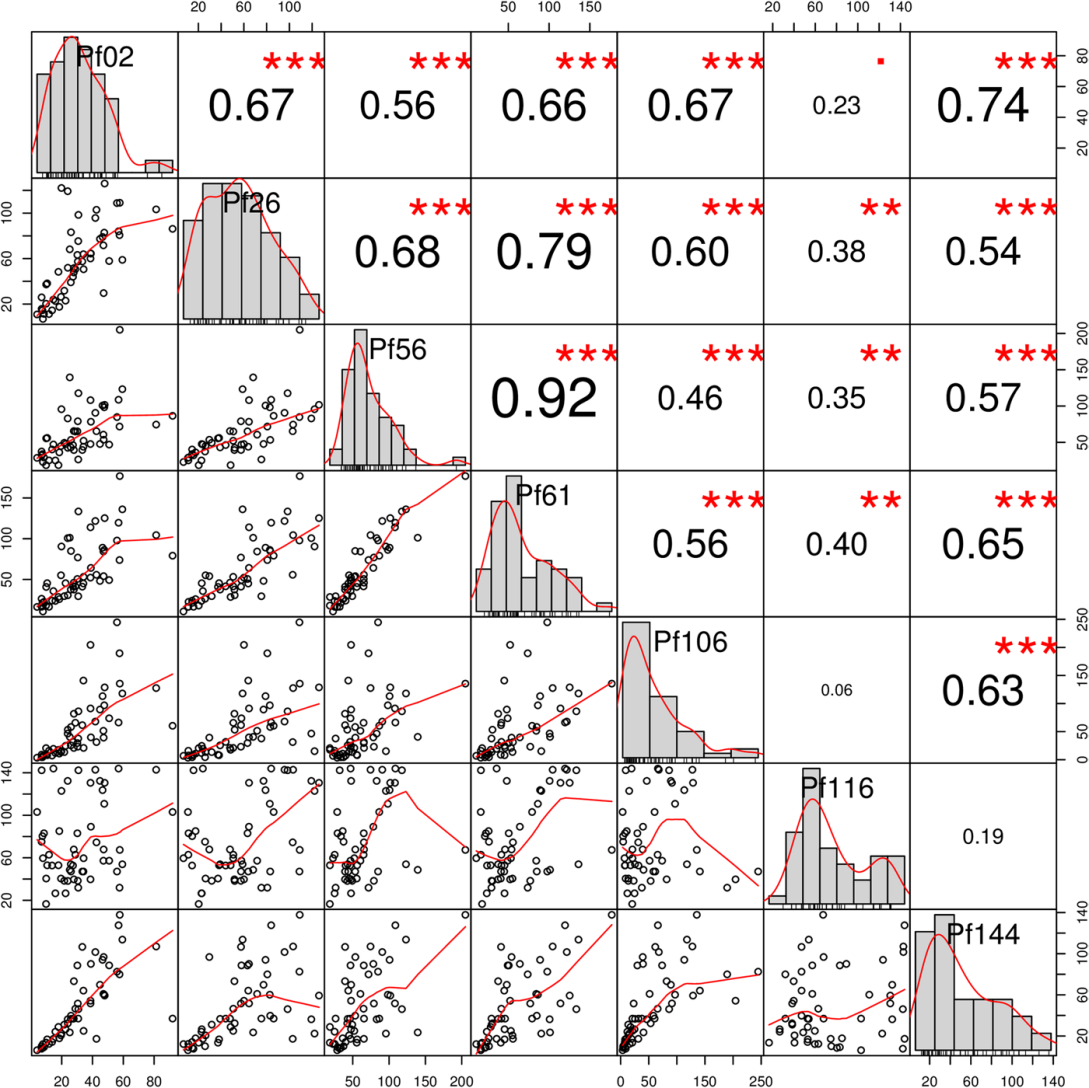
Correlation matrix for pairwise associations between antigen-specific antibody levels. It shows correlation plots in the bottom left part, histograms of antibody titre distribution in the diagonal and the corresponding absolute correlation coefficients and the level of statistical significance (Spearman correlation test) in the upper right part. ^•^Statistical significance at P < 0.05; *Significance at P < 0.01; **Significance at P < 0.001; ***Significance at P < 0.0001.

A correlation matrix was created in order to assess the relationships between the different antigen-specific antibody levels (Fig. 4). There was significant correlation between antigen-specific antibody pairs, with the exception of pairs with anti-Pf116 antibodies, which only showed statistically significant but weak correlation (0.40 or less) with all other antibodies and no significant correlation with anti-Pf106 and anti-Pf144 antibodies (Fig. 4).

### Specific antibodies against the seven PE antigens are not associated with protection against clinical malaria

Antibody data was fitted using a mixed effects model to identify potential antibody correlates of protection against malaria (Table 2). None of the antigen-specific antibodies showed an association with protection from clinical malaria since none of the antibody fixed effects had a statistically significant coefficient. Thus the levels of antibodies to all seven PE antigens, on the basis of the data present here, are most likely markers of exposure to malaria infection but not associated with protection against clinical malaria

**Table 2:** Results of linear fixed effects modeling of antigen-specific antibody data.

Antigen	Fixed effects	Estimate	Standard error	P value (two-tailed)
Pf02	Intercept	16.59	7.36	0.024
	Log_2_(titre)	−0.52	1.30	0.69
Pf26	Intercept	13.35	6.66	0.045
	Log_2_ (titre)	0.11	1.13	0.92
Pf56	Intercept	15.91	13.14	0.23
	Log_2_ (titre)	−0.32	2.28	0.89
Pf61	Intercept	15.01	9.14	0.10
	Log_2_ (titre)	−0.02	1.55	0.990
Pf106	Intercept	13.46	5.23	0.010
	Log_2_ (titre)	0.12	0.91	0.90
Pf116	Intercept	13.64	12.43	0.27
	Log_2_ (titre)	0.20	2.09	0.93
Pf144	Intercept	15.04	6.44	0.019
	Log_2_ (titre)	−0.10	1.14	0.93

## Discussion

Antigen discovery and the identification of immunologically relevant antigens from multiple parasite stages are crucial for designing effective diagnostics and disease monitoring tools as well as for anti-malarial vaccine development. For the latter application, it is currently believed that an effective vaccine must include multiple antigens from different parasite stages^20,21^. Antigens that are expressed in multiple parasite stages are however ideal since immune responses against such single targets could be effective against the multiple stages.

The aim of this study was to assess natural antibody responses to seven *P. falciparum* PE antigens using plasma samples from a longitudinal cohort of young children living in a malaria endemic area of Ghana with seasonal disease transmission. The majority of children were positive for blood film parasites over the entire period of sample collection, but only a limited number of clinical malaria cases were observed. Anaemia was the only condition identified, with the majority of cases occurring during the rainy season (Table 1), most likely related to increased parasite burden. The study data further show that none of the seven antigen-specific antibody responses changed significantly across the six sampling time points when data for all study participants were combined. Though this observation suggests that the plasma levels of antibodies against these antigens are stable throughout the year, it could simply be because the analysis here included data from both parasite-positive and parasite-negative children over the one year study period.

Levels of antibodies against five (Pf02, Pf61, Pf106, Pf116 and Pf144) of the seven antigens were however significantly higher in parasite-positive children, especially during periods of low disease transmission (Fig. 3). The five antigens are all expressed at both the pre-erythrocytic and erythrocytic parasite stages, with the exception of Pf116 which is not expressed in the blood stages but is localized to the surface of pre-erythrocytic stage parasites^17^. The higher antibody responses to these antigens in parasite-positive children during the low transmission period may suggest a direct relationship with the antigen expression levels in the parasite. This is especially so for the antigens that are also found in the erythrocytic stages because of the cyclic nature of the blood stage infection. Of the seven antigens, five are expressed in pre-erythrocytic and asexual blood stage parasites, while Pf26 and Pf116 are expressed in the pre-erythrocytic stages alone^17^. Comparable observations have previously been made in children of similar age for known parasite antigens such as apical membrane antigen 1 (AMA1) and the merozoite surface proteins (MSPs)^12^. The long term availability of these proteins from the cyclic asexual blood stages result in prolonged stimulation of the immune system to elicit antigen-specific antibodies. This may also be true for the pre-erythrocytic antigen Pf116, as it is localized to the sporozoite surface like CSP. Conversely, the absence of re-stimulation of the immune system in parasite-negative children will result in a decrease in malaria antigen-specific antibody levels, as has been previously observed^22-25^. The potential of the five antigens to discriminate between parasite-positive and parasite-negative children during periods of low transmission makes them very essential markers for transmission monitoring. This holds even more relevance for settings where malaria transmission intensity has reduced significantly to the extent that conventional methods of transmission monitoring, such as entomological inoculation rate estimation, have become less sensitive^26^.

Only anti-Pf116 antibody levels showed a moderate negative correlation with parasitaemia at a single time point (November 2004), and the significance of this is not currently apparent. No direct relationship was identified between specific antibody levels on the one hand and the parasite developmental stage specificity, antigen localization or function of the seven putative proteins.

Most of the sampling time point-specific differences in antibody levels between parasitaemic and non-parasitaemic children occurred between January 2005 and March 2005, which coincided with the dry season when malaria transmission was low. Differences in antibody levels between parasite-positive and parasite-negative children were however not generally seen during the major transmission period, especially between July and November 2004 (Fig. 3). These may be explained by more frequent exposure to infectious bites during the rainy season compared to the dry season. Children classified as parasite-negative during the rainy season when transmission intensity was high may thus have had recent infections that had either become sub-microscopic or been cleared at the times of sampling. The slight offset between the climatic and disease transmission seasons has been previously observed and explained^11,27^.

The observations collectively suggest that the measured antibodies are potential markers of the extent of exposure to infectious bites, and this has previously been described for both pre-erythrocytic and erythrocytic stage parasites in this age group of children^12,28,29^. Antibodies in this age group appear to be markers of exposure rather than correlates of protection because their levels are probably below the threshold required to achieve clinical immunity^8,12,30,31^. This threshold may most likely be achieved with age and after several repeat infections^8,12^. The direct association of parasite presence with these antigen-specific antibody levels suggests a potential for these antigens to be used as malaria transmission monitoring biomarkers.

The observation that antigen-specific antibodies measured in this study were exposure biomarkers rather than correlates of clinical protection was confirmed by data from regression analysis, which showed no association between any of the antigen-specific antibody responses and association with clinical protection from either clinical malaria or parasite infection (Table 2). A birth cohort study in Mozambican children reported very similar results of no association between levels of specific antibodies to a number of pre-erythrocytic and erythrocytic stage antigens and increased risk of clinical malaria after nine months of follow up^32^. Subsequent analysis of data after 24 months of follow up showed association of antibody levels against some erythrocytic stage antigens (CyRPA, DBL3_x_, DBL5ε, EBA140_III-V_) with a reduced risk of clinical malaria. This protective effect was however only detected after accounting for heterogeneity in exposure to parasites by including only parasite infected children and considering differences in exposure levels based on clinical, serological and demographic data in Cox regression models^32^. These conditions were met in the current study as two parasite exposure determinants (bednet usage and sampling time point, due to seasonality of disease transmission) as well as age were considered as potential confounders but these did not significantly influence or improve the assessment models. Additionally, assessment of correlates of protection in this study included only data from children who had demonstrable exposure to malaria parasites as assessed by blood film examination. These notwithstanding, it is important to note that while a greater proportion of children were blood film parasite-positive over the six sampling time points, very few children suffered clinical malaria episodes over the same period. This could therefore influence the output from our regression analysis. Some of these antigens are being further investigated as potential vaccine candidates^33^.

Our inability to detect sub-microscopic parasitaemia due to the unavailability of corresponding DNA samples, and the uneven numbers of parasitaemic and non-parasitaemic children from one sampling time point to another are important limitations to the study and our analysis. These notwithstanding, we present very relevant information that adds to the literature on malaria parasite antigens and their potential applications for disease control.

In summary, the data shows the potential of at least five of the seven antigens as biomarkers for monitoring and diagnostics purposes. These antigens may also need to be further investigated for their vaccine potential using a well-defined case-control design in a naturally exposed population.

## Methods

### Ethics statement

Approvals for this study were obtained from the Institutional Review Boards of the Noguchi Memorial Institute for Medical Research (NMIMR, protocol number CPN 029/03-04) and the Navrongo Health Research Centre (NHRC, protocol number NHRCIRB180) in Ghana, as well as from the Ethics Review Committee at the Naval Medical Research Unit 3 (NAMRU-3, protocol number NAMRU3.2014.0004) in Cairo, Egypt. All three ethics bodies have a United States Government Federal Wide Assurance from the Office for Human Research Protections. The original study that collected plasma samples also obtained approvals from the NMIMR and NHRC Ethics Committees stated above. The study was conducted in accordance with the principles in The Belmont Report and federal regulations regarding the protection of human subjects in research. Written informed consent was obtained from parents/guardians of all child subjects before their recruitment and inclusion in the study.

### Study design and samples

**Figure 5:**
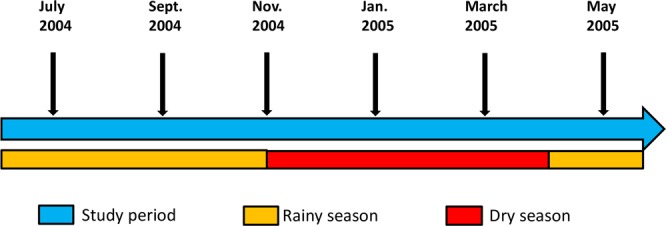
Schematic showing the climatic seasons and sampling time points.

This study utilized stored plasma samples 48 children collected at six time points per child (288 plasma samples in total) in a previous study in the Kassena-Nankana District of the Upper East Region of Ghana. The dry season in the region spanned between November and April of the following year, and the rainy season was between May and October^34,35^. Malaria transmission in this region has been seasonal and overlaps with the rainy and dry seasons. A schematic of the current study design and sampling time points is presented in Fig. 5.

The malaria attack rate in the district during the study period was approximately 3.5 attacks per child per year^36^. Samples originally came from a cohort of 325 children between 1 and 5 years old who were enrolled in May 2004 and followed up every two months until May 2005. Finger-prick blood samples (0.5–1 ml) were collected at the bi-monthly follow-up times for the preparation of plasma. Also at these sampling time points, body temperature was measured and parasite infection status was determined by microscopic examination of thick and thin blood smears. Blood haemoglobin (Hb) levels were measured using a Hemocue^®^ device. Demographic information and data relating to the subjects’ malaria exposure during the transmission season was captured by a study questionnaire. Clinical malaria, for the purposes of this analysis, was defined as having a febrile episode (temperature >37.5 °C), parasite positivity by microscopy and at least one other common symptom of malaria that included malaise, headache, chills, diarrhoea and vomiting. Children diagnosed with clinical malaria were referred to the nearest designated health facility for appropriate treatment as specified in the study protocol. Children who were included in the current study were randomly selected on the basis of having at least one parasite-positive blood smear at any of the six follow up time points and on the availability of samples from the six study time points. For the current study, anaemia was defined as either mild, moderate or severe based on WHO’s standard definitions^37^.

### Antigens and immunological assays

Seven *P. falciparum* antigens were used for the measurement of naturally induced antigen-specific antibody levels. Selection of these antigens was on the basis of their expression in more than one developmental stage of the parasite, and on their recognition by antibodies from RAS-immunized subjects as well as specific antibodies raised in mice and rabbits^17^.

All antigens were expressed in a cell-free wheat germ system as GST-tagged proteins and purified as previously described^17^. The known characteristics of each antigen, including their putative description, the parasite stage specificity and localization within the parasite are presented in Table 3. The seven antigens were selected on the basis of being sporozoite and/or liver PE proteins, but five of them have also been demonstrated to be expressed in the blood stage in localization studies with specific antibodies^17^.

**Table 3:** Characteristics of the seven study antigens.

Antigen	Gene locus	Parasite stage	Spz localization	Functional protein description
Pf02	PFE0785c	All stages	Surface	Glideosome-associated protein 40 (GAP40)*
Pf26	PFI1425w	Pre-erythrocytic	ND	Transcription initiation factor TFIID subunit 7 (TAF7)*
Pf56	PF08_0008	All stages	Surface, cytoplasm	GPI-anchored micronemal antigen (GAMA)
Pf61	PF10_0138	All stages	Cytoplasm	Conserved *Plasmodium* Protein, unknown function
Pf106	PFI0580c	All stages	Interior vesicles	Falstatin, Cysteine Protease Inhibitor (ICP)
Pf116	PFI0460w	Pre-erythrocytic	Surface	Subpellicular microtubule protein 1 (SPM1)*
Pf144	PF14_0467	All stages	Micronemes, ER	Gamete egress and sporozoite traversal protein (GEST)*

*Putative proteins; Spz = sporozoite.

ND = Not determined.

Source: Aguiar *et al*.^17^.

### Measurement of antigen-specific antibody levels

Antigen-specific antibody levels were measured using an enzyme-linked immunosorbent assay (ELISA) approach that involved coating of MaxiSorp^®^ ELISA plates (NUNC, Denmark) with the respective recombinant proteins and incubating overnight at 4 °C. After plates were blocked, plasma samples, diluted 1:100 were incubated and antibody reactivity detected by horseradish peroxidase-conjugated anti-human IgG, followed by incubation with the 3, 3′, 5, 5′-Tetramethylbenzidine (TMB). The color reaction was terminated by addition of 0.2 N H_2_SO_4_ and absorbance read at 450 nm. Absorbance data was converted into titers, expressed in arbitrary units (AU), using the four-parameter logistic curve fitting program known as ADAMSEL (Edmond J. Remarque^®^).

For quality control, a pool of hyperimmune sera (positive control) from malaria exposed adults were run in duplicate on each antigen specific plate and the resulting titer plotted on a Levey-Jennings chart that was generated from previous repeated runs of the same pooled hyperimmune sera. Assay data on any plate was passed only when the positive control on that plate fell within two standard deviations of the mean on the Levey-Jennings chart.

### Data analysis

Antigen-specific antibody levels are presented as box and whisker plots and comparison of antibody levels between parasitaemic and non-parasitaemic children at each sampling time point was performed using the Mann-Whitney U test. The relationship between antigen-specific antibody levels and parameters such as Hb levels and parasitaemia were assessed by Spearman correlation tests. Differences were considered to be statistically significant when P values were less than 0.05. For the assessment of potential antibody correlates of protection against clinical malaria, a generalized mixed effects regression model for repeated measurements was fitted to antigen specific antibody data using the R package *lme4*. For this analysis, the data was categorized into two groups; samples from children who experienced at least one clinical episode over the study period and those from children who remained asymptomatic despite evidence of malaria infection by microscopy at any of the six sampling time points. Initially, antigen-specific antibody data was entered into the model as fixed effects with age as an interaction term, while sampling time point, study subject and bednet usage were entered as random effects. This model was compared to other models that either dropped the subject age interaction term or one of the random effects, and the best model, based on the lowest Akaike Information Criterion (AIC) values was selected for further analysis. The best model had log_2_–transformed antibody level as the fixed effect and the study subject variable as a random effect since the interaction term (age), bednet usage and sampling time point did not significantly contribute to the models. P-values for the intercept and fixed effect were obtained by likelihood ratio tests (with Laplace Approximation).

## Supplementary information

Supplementary file

## Data Availability

All data generated or analysed during this study are included in this published article.
