# Supplementary material for: Antigenicity and immune correlate assessment of seven Plasmodium falciparum antigens in a longitudinal infant cohort from northern Ghana
Source: Sci Rep. 2019 Jun 13;9:8621. doi: 10.1038/s41598-019-45092-4 (PMC6565625; doi:10.1038/s41598-019-45092-4)
Supplement: Supplementary file 1 — Supplementary file [file 41598_2019_45092_MOESM1_ESM.pdf]

Antigenicity and immune correlate assessment of seven *Plasmodium falciparum* antigens in a longitudinal infant cohort from northern Ghana

Kwadwo Asamoah Kusi[1*], Joao Aguiar[2], Selassie Kumordjie[1], Felix Aggor[1#], Jessica Bolton[2], Andrea Renner[2&], Eric Kyei-Baafour[1], Naiki Puplampu[3], Maria Belmonte[2], Daniel Dodoo[1], Ben Adu Gyan[1], Michael Fokuo ofori[1], Abraham Rex Oduro[4], Frank Atuguba[4], Kwadwo Ansah Koram[5], Nehkonti Adams[3^], Andrew Letizia[3], Eileen Villasante[2], Martha Sedegah[2]

Supplementary Table S1: Median, 25th and 75th percentiles for time point specific antibody levels

Antibody	Sampling time point	Median	25th percentile	75th percentile
Anti-Pf02	July. 2004	21.14	15.58	34.11
	Sept. 2004	26	15.23	34.38
	Nov. 2004	24.27	14.23	42.49
	Jan. 2005	27.8	18.27	43.79
	March. 2005	28.99	17.24	54.78
	May. 2005	23.21	13.99	36.95
Anti-Pf26	July. 2004	56.13	31.21	86.94
	Sept. 2004	51.71	26.34	71.47
	Nov. 2004	46.88	22.81	106.27
	Jan. 2005	64.49	39.12	82.53
	March. 2005	54.85	38.93	84.04
	May. 2005	44.03	31.08	63.26
Anti-Pf56	July. 2004	39.43	24.49	57.91
	Sept. 2004	42.79	24.07	53.93
	Nov. 2004	47.78	29.31	72.19
	Jan. 2005	44.47	34.4	99.2
	March. 2005	53.63	35.19	70.05
	May. 2005	40.22	25.75	55.7
Anti-Pf61	July. 2004	47.74	23.15	89.2
	Sept. 2004	55.17	30.65	79.92
	Nov. 2004	58.76	28.77	97.84
	Jan. 2005	57.44	36.7	103.07
	March. 2005	68.18	29.46	113.46
	May. 2005	49.38	24.66	83.5
Anti-Pf106	July. 2004	38.03	19.23	83.58

	Sept. 2004	37.72	21.12	78.62
	Nov. 2004	46.12	17.58	87.24
	Jan. 2005	38.81	28.96	117.3
	March. 2005	39.36	25.65	91.42
	May. 2005	39.08	18.38	56.98
Anti-Pf116	July. 2004	54.29	30.8	136.22
	Sept. 2004	48.68	38.26	83.65
	Nov. 2004	67.44	37.24	111.85
	Jan. 2005	74.55	41.05	120.68
	March. 2005	85.91	37.77	142.85
	May. 2005	57.27	36.62	83.12
Anti-Pf144	July. 2004	23.15	14.98	40.22
	Sept. 2004	25.7	13.6	48.03
	Nov. 2004	22.22	13.53	54.19
	Jan. 2005	36.6	17.73	70.31
	March. 2005	38.92	17.31	94.05
	May. 2005	22.4	14.23	49.9

Supplementary Table S2: Comparison of time point specific antibody levels between parasitaemic and non-parasitaemic children

Antibody	Sampling time point	Parasitaemic			Non-parasitaemic			P value
		Median	25th percentile	75th percentile	Median	25th percentile	75th percentile	
Anti-Pf02	July. 2004	23.32	17.36	34.52	11.33	9.78	13.33	**0.007**
	Sept. 2004	27.04	16.16	34.47	16.93	10.88	29.99	0.39
	Nov. 2004	25.18	16.34	50.65	10.24	7.465	23.425	0.053
	Jan. 2005	29.07	20.61	45.92	14.17	9.29	32.78	0.068
	March. 2005	42.66	24.53	57.74	11.14	7.84	21.05	**< 0.0001**
	May. 2005	28.25	20.08	39.32	11.9	8.415	26.26	**0.026**
Anti-Pf26	July. 2004	48.37	30.45	83.19	69.92	50.7	103.96	0.52
	Sept. 2004	51.71	24.12	69.83	54.98	46.13	63.83	0.82
	Nov. 2004	66.99	25.81	108.93	19.25	17.51	21	0.16
	Jan. 2005	64.49	40.69	80.64	82.59	60.85	104.33	0.64
	March. 2005	79.07	50.61	87.34	43.38	29.2	48.23	0.06
	May. 2005	47.39	32.53	61.8	33.88	31.22	73.27	1
Anti-Pf56	July. 2004	41.41	22.4	57.32	30.84	27.39	50.33	0.93

	Sept. 2004	42.56	23.11	53.36	43.02	37.2	49.28	0.72
	Nov. 2004	58.64	36.22	78.44	25.11	24.99	28.61	0.082
	Jan. 2005	44.47	33.63	93.39	72.4	57.75	87.05	0.67
	March. 2005	58.62	47.14	78.17	31.98	28.17	53.11	0.052
	May. 2005	46.34	29.17	63	32.67	22.67	45.78	0.19
Anti-Pf61	July. 2004	49.38	26.33	90.62	46.2	15.91	47.27	0.60
	Sept. 2004	55.17	30.65	78.06	60.38	35.44	92.5	0.75
	Nov. 2004	61.92	37.87	99.95	17.93	16.91	50.73	0.093
	Jan. 2005	64.2	40.41	114.02	15.97	14.36	57.45	**0.029**
	March. 2005	89.03	53.79	121.76	19.27	13.11	53.95	**< 0.0001**
	May. 2005	59.35	36.15	86.08	29	15.1	54.34	0.067
Anti-Pf106	July. 2004	42.62	19.79	89.34	23.19	16.62	32.14	0.22
	Sept. 2004	38.37	21.21	83.47	27.18	16.82	38.08	0.19
	Nov. 2004	50.11	26.32	95.5	16.64	12.14	27.68	0.099
	Jan. 2005	54.26	31.25	122.23	22.1	17.22	28.66	**0.016**
	March. 2005	59.23	30.61	96.05	21.03	12.91	32.21	**0.0071**
	May. 2005	44.15	30.55	72.11	18.38	14.85	51.44	**0.017**
Anti-Pf116	July. 2004	55.59	43.66	147.78	28.09	24.5	51.62	0.19
	Sept. 2004	48.77	38.26	102.47	42.16	41.33	42.99	0.67
	Nov. 2004	74.57	47.12	107.47	32.49	26.89	116.09	0.32
	Jan. 2005	83.35	53.93	130.55	28.64	26.68	48.02	**0.019**
	March. 2005	113.29	80.39	143.69	29.66	24.86	81.82	**0.018**
	May. 2005	62.89	52.75	83.69	35.92	27.57	61.48	0.053
Anti-Pf144	July. 2004	27.61	13.56	40.45	21.56	18.45	23.09	0.93
	Sept. 2004	26.41	14.95	50.6	13.55	9.575	24.795	0.25
	Nov. 2004	27.74	17.08	63.45	7.72	7.56	19.09	**0.03**
	Jan. 2005	39.18	18.62	89.12	22.32	16.11	33.61	0.22
	March. 2005	76.99	42.72	101.27	17.57	11.12	22.2	**0.0009**
	May. 2005	36.38	18.58	53.94	19.71	13.27	31.11	0.13

Footnotes:
P values from Mann-Whitney U tests
